# Machine learning-based radiomics prognostic model for patients with proximal esophageal cancer after definitive chemoradiotherapy

**DOI:** 10.1186/s13244-024-01853-y

**Published:** 2024-11-29

**Authors:** Linrui Li, Zhihui Qin, Juan Bo, Jiaru Hu, Yu Zhang, Liting Qian, Jiangning Dong

**Affiliations:** 1 Department of Radiation Oncology, Anhui Provincial Hospital Affiliated to Anhui Medical University, Hefei, China; 2 Department of Radiation Oncology, West Branch of the First Affiliated Hospital of University of Science and Technology of China, Hefei, China; 3 Department of Radiation Oncology, The First Affiliated Hospital of Anhui Medical University, Hefei, China; 4 Department of Radiology, West Branch of the First Affiliated Hospital of University of Science and Technology of China, Hefei, China

**Keywords:** Radiomics, Machine learning, Proximal esophageal cancer, Tumor microenvironment

## Abstract

**Objectives:**

To explore the role of radiomics in predicting the prognosis of proximal esophageal cancer and to investigate the biological underpinning of radiomics in identifying different prognoses.

**Methods:**

A total of 170 patients with pathologically and endoscopically confirmed proximal esophageal cancer from two centers were enrolled. Radiomics models were established by five machine learning approaches. The optimal radiomics model was selected using receiver operating curve analysis. Bioinformatics methods were applied to explore the potential biological mechanisms. Nomograms based on radiomics and clinical–radiomics features were constructed and assessed by receiver operating characteristics, calibration, and decision curve analyses net reclassification improvement, and integrated discrimination improvement evaluations.

**Results:**

The peritumoral models performed well with the majority of classifiers in the training and validation sets, with the dual-region radiomics model showing the highest integrated area under the curve values of 0.9763 and 0.9471, respectively, and outperforming the single-region models. The clinical–radiomics nomogram showed better predictive performance than the clinical nomogram, with a net reclassification improvement of 34.4% (*p* = 0.02) and integrated discrimination improvement of 10% (*p* = 0.007). Gene ontology enrichment analysis revealed that lipid metabolism-related functions are potentially crucial in the process by which the radiomics score could stratify patients.

**Conclusions:**

A combination of peritumoral radiomics features could improve the predictive performance of intratumoral radiomics to estimate overall survival after definitive chemoradiotherapy in patients with proximal esophageal cancer. Radiomics features could provide insights into the lipid metabolism associated with radioresistance and hold great potential to guide personalized care.

**Critical relevance statement:**

This study demonstrates that incorporating peritumoral radiomics features enhances the predictive accuracy of overall survival in proximal esophageal cancer patients after chemoradiotherapy, and suggests a link between radiomics and lipid metabolism in radioresistance, highlighting its potential for personalized treatment strategies.

**Key Points:**

- Peritumoral region radiomics features could predict the prognosis of proximal esophageal cancer.
- Dual-region radiomics features showed significantly better predictive performance.
- Radiomics features can provide insights into the lipid metabolism associated with radioresistance.

**Graphical Abstract:**

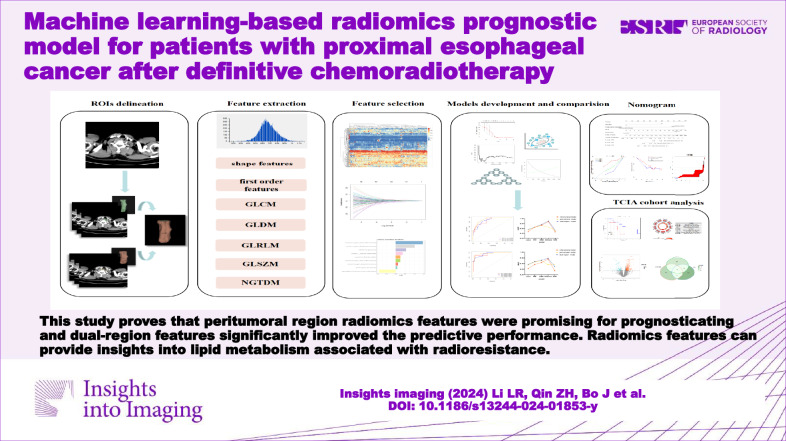

## Introduction

Esophageal cancer (ESCA) is one of the most common malignancies worldwide, ranking seventh in incidence and sixth in mortality [1]. Proximal ESCA, which includes tumors in the cervical and upper-thoracic esophageal sections, occurs rarely and accounts for approximately 10% of all esophageal carcinomas [2]. However, patients with proximal ESCA have a poor prognosis, with a 5-year overall survival (OS) rate of only 35–45% [3], which may be because the biological behavior of ESCA at this location is more similar to that of hypopharyngeal carcinoma—a more aggressive type of cancer [4].

Surgery, the primary treatment modality for ESCA, may not always be the best option for proximal ESCA due to the high risk of adjacent anatomical structure invasion and the need for a total laryngopharynx oesophagectomy with reconstruction, which often substantially affects the patient’s quality of life. Therefore, definitive chemoradiotherapy (dCRT) is the current standard of care for proximal ESCA [5, 6]. However, despite rapid advances in radiotherapy techniques, more than 50% of the patients show local and distant recurrence [7, 8], leading to an unsatisfactory prognosis of proximal ESCA. Moreover, patients with the same stage and similar treatment regimens are likely to experience different adverse events, including locoregional recurrence or distant metastasis. Hence, a practical and robust approach for precise pretreatment prediction of clinical outcomes for proximal ESCA patients with different risk stratifications is urgently needed.

Radiomics, as an emerging technique applied in medicine, aims to extract a vast amount of high-dimensional quantitative features that reflect tumor heterogeneity and potential pathophysiological information from a series of medical images [9] and has been widely applied as a cost-effective and efficient method for tumor discrimination and diagnosis, prognosis prediction, treatment evaluation, and even determination of molecular subtypes [10–13]. In recent years, combinations of radiomics data with machine learning algorithms have been used for predicting prognostic information in patients of various cancers, such as melanoma [14], small cell lung cancer [15], and colorectal cancer [16]. Nevertheless, few studies have investigated the prognostic value of radiomics features in OS estimation for patients with in patients with proximal ESCA, and most previous studies have focused on the primary tumor area alone, overlooking the radiomics features derived from the peritumoral area, which are likely to provide more pivotal information characterizing tumor heterogeneity or even the tumor microenvironment (TME). Radiomics features from the peritumoral area have also been shown to be predictive in other cancers. Khorrami et al revealed that the radiomics texture features extracted from around the tumor were predictive of OS in patients with non-small cell lung cancer [17]. Another study by Braman et al showed that peritumoral radiomics features from pretreatment breast dynamic contrast-enhanced-magnetic resonance imaging could successfully predict a pathologic complete response to neoadjuvant chemotherapy [18]. However, to the best of our knowledge, no previous studies have integrated intratumoral and peritumoral radiomics features to predict survival outcomes after definitive chemoradiotherapy for ESCA, especially proximal ESCA.

In this study, we sought to establish different machine-learning-based radiomics models to predict OS in patients with proximal ESCA and assess whether the peritumoral region could provide incremental prognostic value. In addition, we constructed a radiomics–clinical nomogram by integrating the radiomics model with clinical risk factors and explored the possible biological underpinning of radiomics to identify different prognoses.

## Materials and methods

### Study design and patients

This bicentric study was approved by the institutional review boards of centers 1 and 2, and the requirement for written informed consent was waived due to the retrospective nature of the study. A total of 170 eligible candidate patients with proximal ESCA from two centers between January 2015 and December 2020 were included in accordance with the inclusion and exclusion criteria. The inclusion criteria were as follows: (a) patients with histologically and endoscopically confirmed proximal ESCA; (b) underwent definitive chemoradiotherapy or radiotherapy alone; (c) contrast-enhanced computed tomography (CT) scan was performed within 2 weeks before treatment; and (d) clinical data were available. Exclusion criteria were as follows: (a) Poor CT image quality or the tumor lesion was too small to recognize; (b) any treatment history before definitive chemoradiotherapy; (c) combined with other primary tumors; (d) incomplete clinical information; or (e) lost to follow-up.

**Fig. 1:**
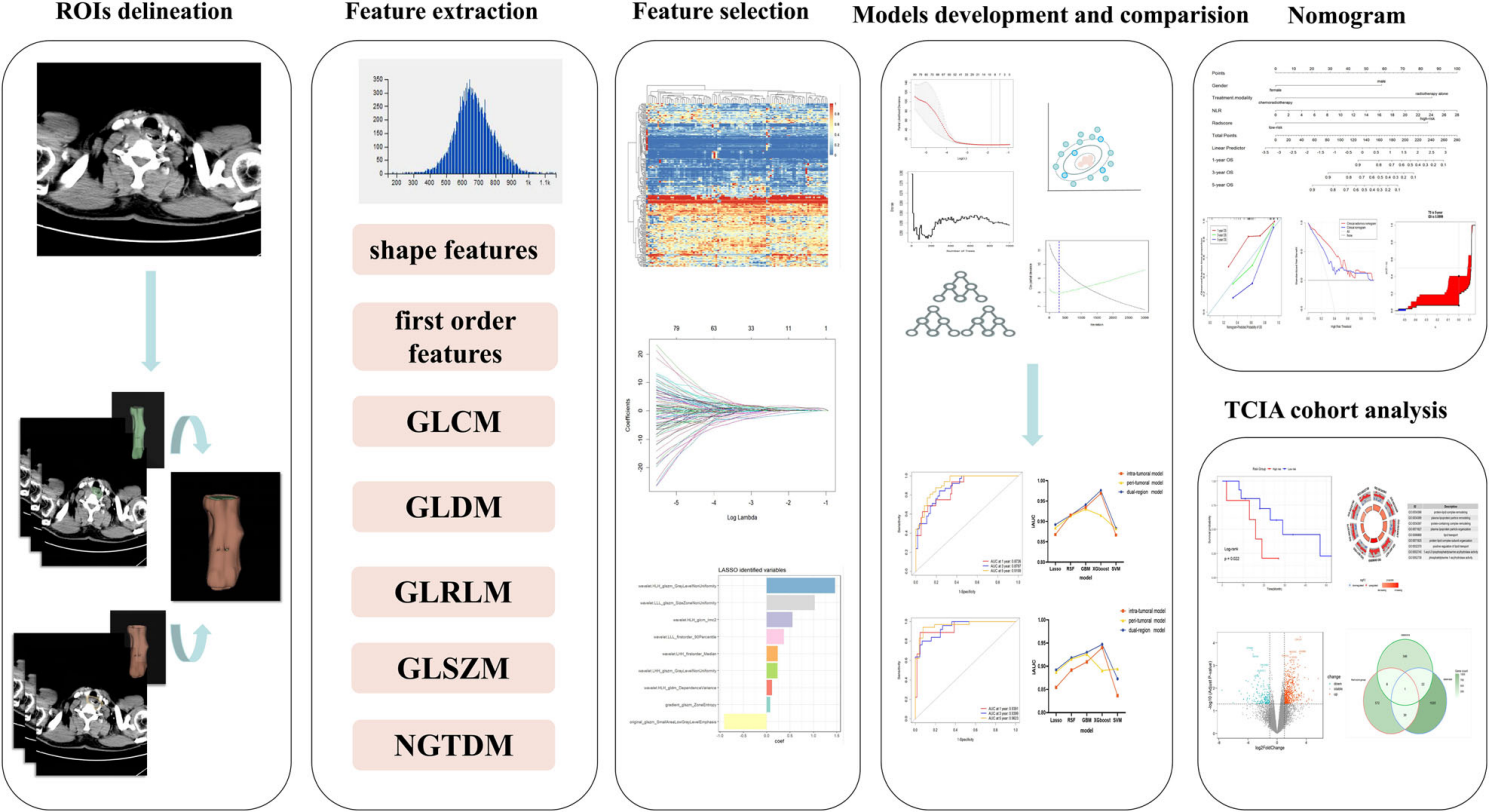
Workflow of this study

Patients from centers 1 and 2 were included in the training and validation cohorts, respectively. The patient selection process is outlined in Supplementary Fig. 1. Another external validation cohort (TCGA/TCIA-ESCA cohort) was collected from the cancer imaging archive (TCIA). ESCA cases in the TCGA/TCIA-ESCA cohort that lacked pretreatment-enhanced CT data were excluded. Eventually, 16 cases were enrolled. The microarray gene expression data, clinical information, and OS outcomes of these 16 patients were downloaded from The cancer genome atlas (TCGA). Figure 1 depicts the overall workflow of this study.

### Treatment and follow-up management

The majority of patients received dCRT, while radiotherapy alone was administered to patients with advanced age or poor performance status, and those who requested radiotherapy alone. The endpoint of this study was OS, which was defined as the interval between the pathological diagnosis and the last follow-up or death. All patients were followed up until death or October 2023, and the median follow-up time in the training and validation cohorts was 56 months and 58 months, respectively. The detailed treatment and follow-up programs are shown in the Supplementary Methods.

### Region of interest delineation and feature extraction

The CT image acquisition parameters are listed in Supplementary Table 1. The anonymized arterial-phase enhanced CT images were imported into 3D-Slicer software from the Picture Archiving and Communication Systems. The regions of interest (ROIs) of the images were manually delineated layer-by-layer and included the intratumoral and peritumoral regions, which were annotated as the area where intratumoral ROIs flared out by 5 mm (Supplementary Fig. 2). All ROIs were fused into the volume of interest (VOI) to extract radiomics features. To account for the feature robustness against intra- and interobserver variations in delineation, we further conducted a test–retest study and determined the intra- and interclass correlation coefficients (ICCs) to assess the reproducibility. Specific details are provided in the Supplementary Methods.

Radiomics features were extracted using an open-source Python tool named PyRadiomics. The feature extraction procedures matched the benchmarks of the image biomarker standardization initiative [19]. A total of 1409 quantitative radiomics features were extracted from each VOI of each patient. The radiomics features collected from the three cohorts were first harmonized using ComBat to minimize the batch effect caused by different CT scanners. In addition, image feature data were standardized and normalized using *z*-score for further analysis.

### Feature selection and radiomics signature models development

To reduce the dimensionality of features, a three-step feature selection strategy was conducted in the training set. First, repeatable features with ICCs > 0.75 were retained for subsequent analysis. Second, Spearman correlation analysis was applied to select features with the least redundancy, and features with correlations greater than 0.8 were removed. Lastly, the remaining features were entered into a least absolute shrinkage and selection operator (LASSO) Cox regression model with 10-fold cross-validation to identify the most predictable features.

Then, the most useful prognostic features were applied to construct radiomics models. Five mainstream machine learning algorithms, such as LASSO—Cox regression, random survival forest (RSF), gradient boosting machine (GBM), extreme gradient boosting (XGboost), and support vector machine (SVM) were chosen as our classifiers to identify an optimal classifier model and a Rad-score that was greatly competent to recognize the prognosis of patients.

### Establishment and validation of clinical and clinical–radiomics nomograms

To verify the incremental value of the radiomics signature beyond the clinical factors, we developed a clinical nomogram alone and a clinical–radiomics nomogram that combined the Rad-score with independent clinical prognostic factors. For the clinical nomogram based on 21 clinical risk factors (including age; gender; tobacco use; alcohol use; cT stage; cN stage; histological type; treatment modality; radiation dose; neutrophil, lymphocyte, monocyte, and platelet counts; hemoglobin and albumin levels; neutrophil–lymphocyte ratio [NLR], platelet–lymphocyte ratio [PLR], and monocyte–lymphocyte ratio [MLR]; and the systemic inflammation response index [SIRI], aggregate index of systemic inflammation [AISI], and prognostic nutritional index [PNI]), univariate Cox analysis was initially used to detect the associations between each factor and the patients’ OS, the significant factors (*p* < 0.1) were entered into the multivariate analysis, and independent clinical prognostic factors (*p* < 0.05) associated with OS were obtained to establish a clinical nomogram. The performances of the nomograms were evaluated and validated in the training and the validation cohorts by using receiver operating characteristic (ROC), calibration, and decision curve analyses and measuring the net reclassification improvement (NRI) and integrated discrimination improvement (IDI).

### Analysis of the association between the Rad-score and the TME

To further explore the biological basis of prognostic prediction based on the Rad-score, the TCGA/TCIA-ESCA cohort was used to further validate the ability of the Rad-score to perform risk stratifications. The same Rad-score acquisition process was applied, and the same cutoff value was used to divide the cohort into low- and high-score groups. Kaplan–Meier survival analysis was performed to explore whether the Rad-score was associated with OS. Then, differentially expressed genes (DEGs) between the high- and low-score groups were identified, and gene ontology (GO) enrichment analyses were conducted. We hypothesized that the Rad-score could predict prognosis by reflecting the TME that leads to radiotherapy resistance. The GSE61816 and GSE61620 datasets downloaded from the Gene Expression Omnibus (GEO) database were used to test this hypothesis. Next, we identified the intersection of the upregulated genes from the three difference analyses to obtain the core genes.

### Statistical analysis

The normality of continuous variables among the baseline characteristics was examined using the Kolmogorov–Smirnov test, and variables showing a normal distribution were compared using the two independent sample *t*-tests, while other continuous variables were compared using the Mann–Whitney *U*-test. Categorical variables were evaluated using the chi-square test or Fisher’s exact probability test. The optimum cutoff value of the Rad-score was determined by using an X-tile. Survival results were compared using Kaplan–Meier analysis and the log-rank test. Performance was assessed with ROC, calibration, and decision curve analyses, as well as by measuring the NRI and IDI. Software and R packages for statistical analyses are provided in Supplementary Methods. A two-tailed *p*-value < 0.05 indicated statistical significance.

## Results

### Baseline characteristics

A total of 170 patients were enrolled in this study, including 97 in the training cohort and 73 in the validation cohort. The patients’ characteristics are presented in Table 1. The majority of the patients were male (> 60%), had cT3- or cT4-stage disease (> 70%), and received dCRT (> 80%). The two cohorts showed no significant differences in terms of age; gender; tobacco use; alcohol use; cT and cN stages; histological type; treatment modality; radiation dose; neutrophil, lymphocyte, monocyte, and platelet counts; hemoglobin and albumin levels; and the NLR, PLR, MLR, SIRI, AISI, and PNI, as shown in detail in Table 1. The median OS was 46 months (16–70.5) in the training cohort and 47 months (20–70) in the validation cohort.

**Table 1:** Patient characteristics in training and validation sets

Characteristic	Training set, (*n* = 97)	Validation set, (*n* = 73)	*p* value
Age, (year)	70 (62, 77)	69 (66, 74)	0.559
Gender			0.767
Male	63 (64.9%)	49 (67.1%)	
Female	34 (35.1%)	24 (32.9%)	
Tobacco use			0.559
Never	78 (80.4%)	56 (76.7%)	
Former or current	19 (19.6%)	17 (23.3%)	
Alcohol use			0.618
Never	80 (82.5%)	58 (79.5%)	
Former or current	17 (17.5%)	15 (20.5%)	
cT stage			0.060
T1	2 (2.1%)	1 (1.4%)	
T2	12 (12.4%)	19 (26.0%)	
T3	66 (68.0%)	37 (50.7%)	
T4	17 (17.5%)	16 (21.9%)	
cN stage			0.164
N0	20 (20.6%)	21 (28.8%)	
N1	43 (44.3%)	21 (28.8%)	
N2	29 (29.9%)	24 (32.9%)	
N3	5 (5.2%)	7 (9.6%)	
Histological type			0.393
Squamous cell carcinoma	93 (95.9%)	72 (98.6%)	
Adenocarcinoma	4 (4.1%)	1 (1.4%)	
Treatment modality			0.321
Radiotherapy alone	11 (11.3%)	5 (6.8%)	
Chemoradiotherapy	86 (88.7%)	68 (93.2%)	
Radiation dose, (Gy)			0.691
≤ 50.4	21 (21.6%)	18 (24.7%)	
> 50.4 Gy ≤ 60	62 (63.9%)	42 (57.5%)	
> 60	14 (15.5%)	13 (17.8%)	
Neutrophil (× 10^9^ L^−^^1^)	3.86 (2.56, 4.95)	3.84 (3.06, 4.72)	0.813
Lymphocyte (× 10^9^ L^−^^1^)	1.14 (0.94, 1.57)	1.05 (0.74, 1.46)	0.282
Monocyte (× 10^9^ L^−^^1^)	0.46 (0.36, 0.59)	0.49 (0.37, 0.68)	0.360
Platelet (× 10^9^ L^−^^1^)	196.03 ± 74.32	215.88 ± 70.93	0.081
Hemoglobin, (g/L)	115.74 ± 17.53	1118.58 ± 19.17	0.317
Albumin, (g/L)	39.81 (35.80, 43.70)	40.70 (37.75, 43.40)	0.324
NLR	3.20 (1.97, 4.22)	2.90 (2.10, 3.94)	0.342
PLR	153.29 (108.04, 214.23)	137.25 (105.36, 194.17)	0.327
MLR	0.39 (0.27, 0.57)	0.33 (0.24, 0.45)	0.096
SIRI	1.45 (0.92, 2.29)	1.38 (0.81, 2.15)	0.473
AISI	288.05 (137.29, 436.99)	225.00 (121.49, 393.11)	0.133
PNI	46.23 ± 7.23	46.67 ± 8.08	0.708
OS, (month)	46 (16, 70.5)	47 (20, 70)	0.985

*NLR* neutrophil to lymphocyte ratio, *PLR* platelet to lymphocyte ratio, *MLR* monocyte to lymphocyte ratio, *SIRI* systemic inflammatory response index, *AISI* aggregate index of systemic inflammation, *PNI* prognostic nutritional index, *OS* overall survival

### Radiomics models and performance

#### Radiomics feature selection

A total of 1409 radiomics features were extracted from a single VOI. The extracted radiomics features were classified into 14 shape features, 270 first-order intensity features, 360 gray level co-occurrence matrix (GLCM), 240 gray level dependence matrix (GLDM), 240 gray level run-length matrix (GLRLM), 210 gray level size zone matrix (GLSZM), and 75 neighbors gray-tone difference matrix (NGTDM) features. After the test–retest experiment, 1206 of the 1409 intratumoral features and 890 of the 1409 peritumoral features showed inter- and intra-ICC (inter- and intraclass correlation coefficient) values > 0.75. Then, through Spearman correlation analysis, 216 and 277 features with the least redundancy were identified. Next, LASSO-Cox regression was used to downscale and select the most valuable features, resulting in 7, 9, and 11 features with non-zero coefficients for the intratumoral, peritumoral, and dual-region signatures, respectively (Table 2).

**Table 2:** Selected radiomics features for different regions

Radiomics model	Selected features
Intra-tumoral model	Intra-wavelet.HHH_glszm_ZoneVariance
	Intra-wavelet.LHH_glszm_SizeZoneNonUniformity
	Intra-wavelet.LLH_glszm_GrayLevelNonUniformity
	Intra-wavelet.LHL_glcm_Correlation
	Intra-wavelet.HLH_glszm_SizeZoneNonUniformity
	Intra-gradient_ngtdm_Busyness
	Intra-lbp.2D_firstorder_Variance
Peri-tumoral model	Peri-wavelet.HLH_glszm_GrayLevelNonUniformity
	Peri-wavelet.LLL_glszm_SizeZoneNonUniformity
	Peri-wavelet.HLH_glcm_Imc2
	Peri-wavelet.LLL_firstorder_90Percentile
	Peri-wavelet.LHH_firstorder_Median
	Peri-wavelet.LHH_glszm_GrayLevelNonUniformity
	Peri-wavelet.HLH_gldm_DependenceVariance
	Peri-gradient_glszm_ZoneEntropy
	Peri-original_glszm_SmallAreaLowGrayLevelEmphasis
Dual-region model	Intra-wavelet.HHH_glszm_ZoneVariance
	Peri-wavelet.LLL_glszm_SizeZoneNonUniformity
	Peri-wavelet.HLH_glszm_GraylevelNonUniformity
	Peri-wavelet.LHH_glszm_GrayLevelNonUniformity
	Peri-wavelet.HLH_glcm_Imc2
	Peri-wavelet.LLL_firstorder_90Percentile
	Intra-wavelet.LHL_glcm_Correlation
	Peri-gradient_glszm_ZoneEntropy
	Peri-wavelet.HLH_gldm_DependenceVariance
	Intra-lbp.2D_firstorder_Variance
	Peri-original_glszm_SmallAreaLowGrayLevelEmphasis

### Radiomics model development and comparison

**Fig. 2:**
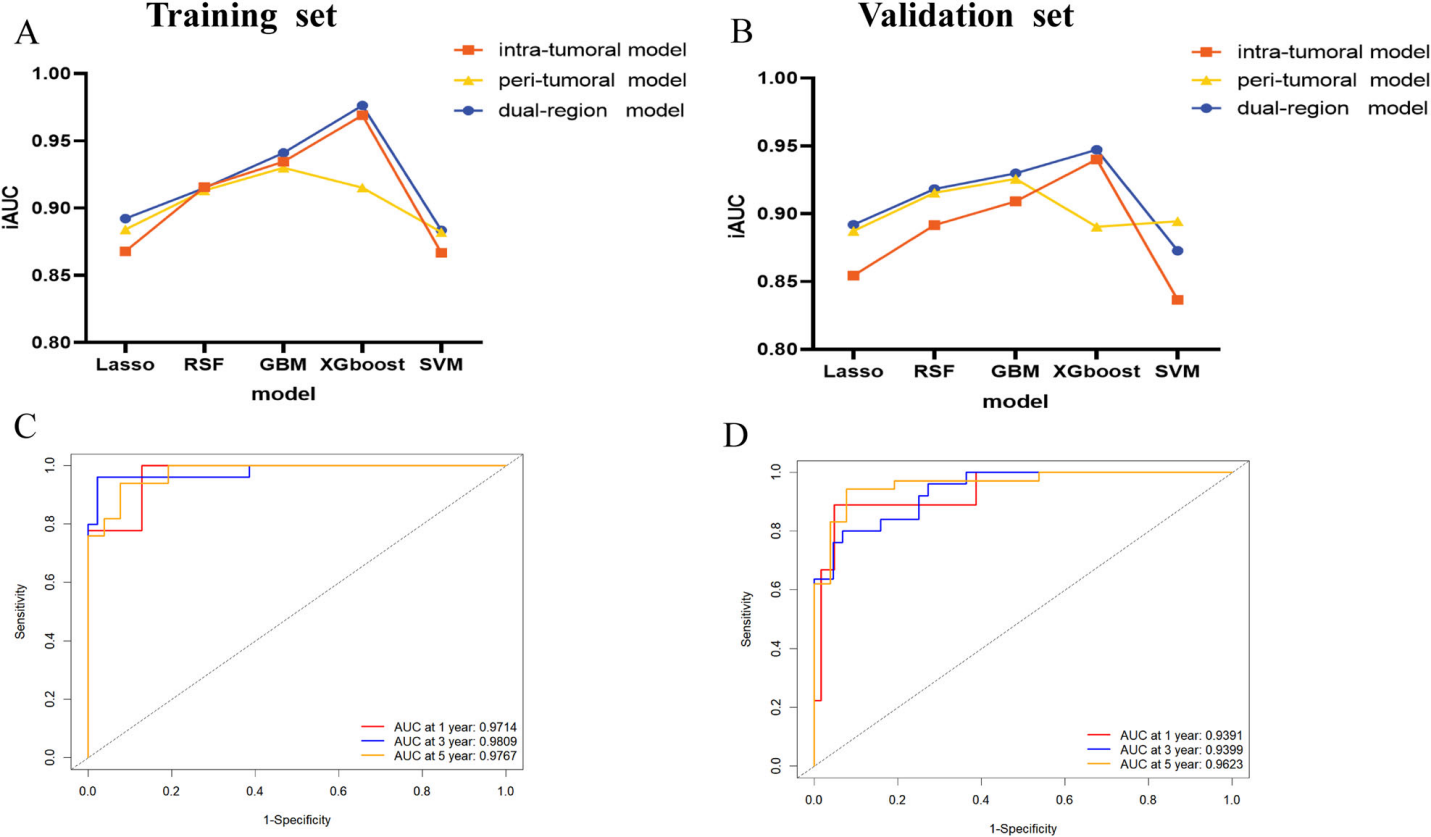
The iAUC of ROC curves in LASSO, RSF, GBM, XGboost, and SVM among intra-, peri-, and dual-regions in the training cohort (**A**) and validation cohort (**B**). The iAUC value was calculated by the mean 1-, 3-, and 5-AUC of ROC curves, respectively. ROC curves for 1-, 3-, and 5-year survival prediction using optimal radiomics model (XGboost in dual-region) in the training cohort (**C**) and validation cohort (**D**). iAUC, the integrated area under the curve; ROC, receiver operating characteristic

The ROC curves of 1-, 3-, and 5-year OS prediction using intratumoral, peritumoral, and dual-region features among the five classifiers are shown in Supplementary Figs. 3–5. We used the integrated area under the curve (iAUC) to compare performances across various classifiers and different regions (Fig. 2A, B). The XGboost classifier exhibited the highest iAUCs for intratumoral (iAUC = 0.9400) and dual-region (iAUC = 0.9471) models in the validation cohort, whereas GBM demonstrated the best performance for the peritumoral model, achieving an iAUC of 0.9257. Moreover, the peritumoral model performed well with iAUCs > 0.9 for the majority of classifiers in the training and validation cohorts, indicating that radiomics features from the peritumoral area play a crucial role in prognostic prediction. The dual-region model outperformed the single-region models for most classifiers in both the training and validation sets, while XGboost achieved the best performance with iAUCs of 0.9763 and 0.9471, respectively. Therefore, we finally chose XGboost with a dual-region radiomics model as the optimal model for further analysis and calculated its Rad-score for each patient. The ROC curves of the optimal model (XGboost in dual-region) are shown in Fig. 2C, D.

### Incremental value of the Rad-score

**Fig. 3:**
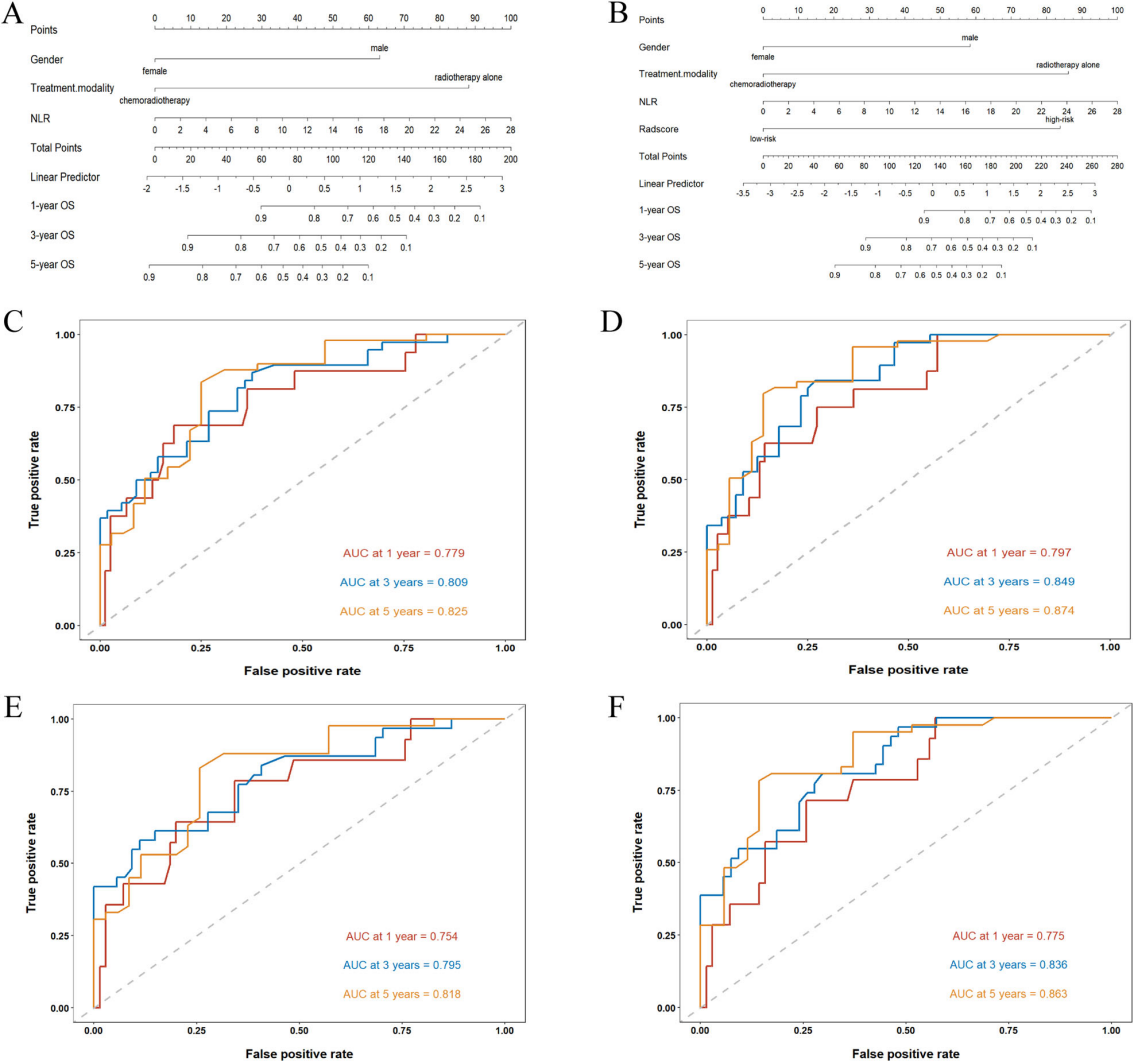
Nomograms for OS prediction of clinical nomogram (**A**) and clinical–radiomics nomogram (**B**). The ROC curves of clinical nomogram and clinical–radiomics nomogram in training set (**C**, **D**) and validation set (**E**, **F**). ROC, receiver operating characteristic

**Fig. 4:**
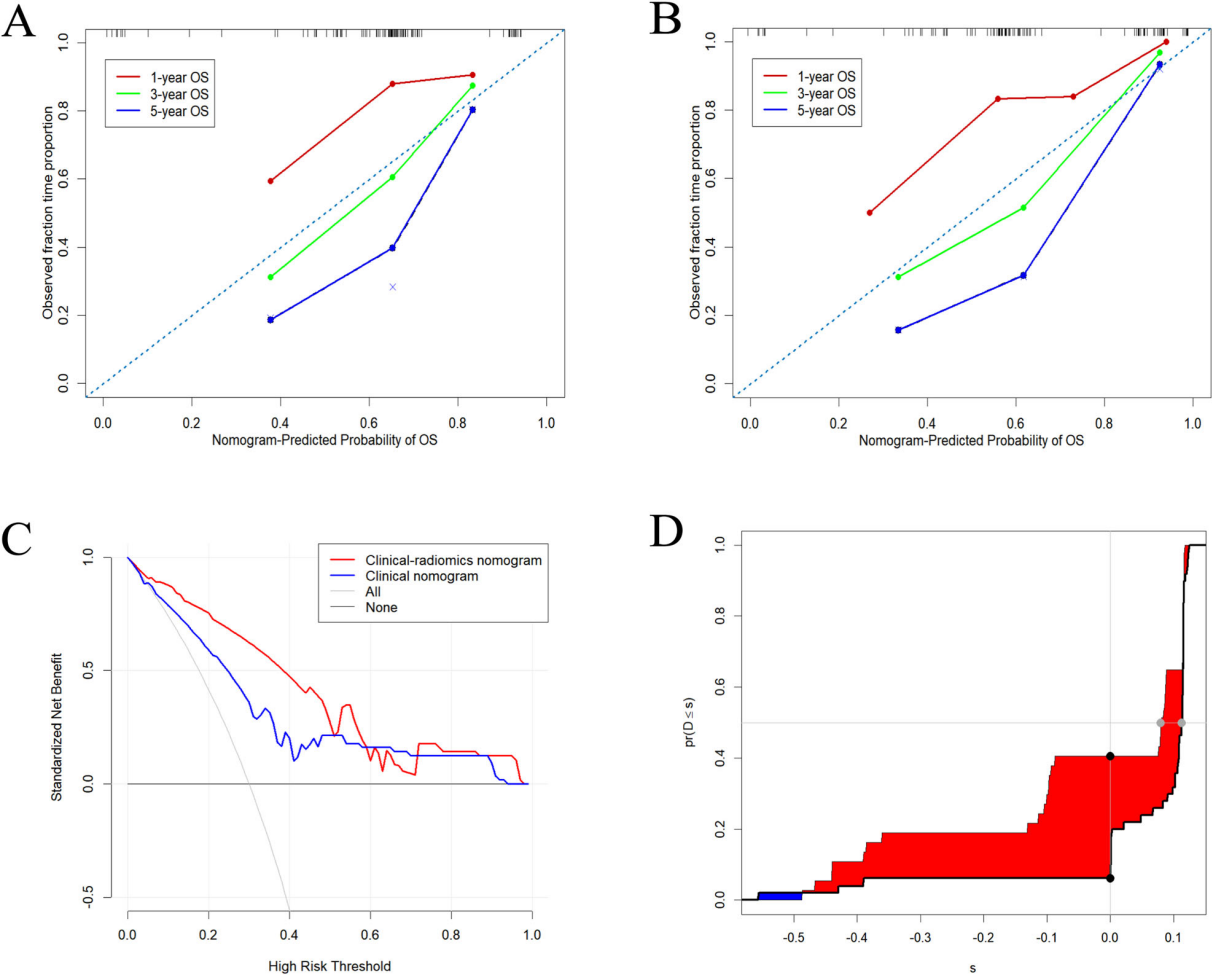
Calibration curve of clinical nomograms (**A**) and clinical–radiomics nomograms (**B**). The dotted line represents the ideal nomogram, while the red/green/blue line represents the observed line for 1/3/5 year, respectively. Decision curve analysis (**C**). The *y*-axis measures the net benefit. The net benefit was calculated by summing the benefits (true positive results) and subtracting the harms (false positive results), weighting the latter by a factor related to the relative harm of undetected cancer compared with the harm of unnecessary treatment. The net benefit of the decision based on clinical–radiomics nomogram (red line) is superior to the other three for OS of proximal ESCA. Performance improvement evaluation of different nomograms for OS with IDI (**D**)

After screening the clinical factors, gender, treatment modality, and NLR were identified as independent predictors of OS in the training set by univariable and multivariable Cox regression analysis (Table 3). The patients were classified into a high-risk group (Rad-score > 14.460) and a low-risk group (Rad-score < 14.460) on the basis of the optimal cutoff value generated by the X-tile. Subsequently, the clinical and clinical–radiomics nomograms for predicting the prognosis based on OS were constructed and are presented in Fig. 3A, B. The ROC curves of the two nomogram models in the training and validation cohorts are also presented in Fig. 3C–F. The clinical–radiomics nomogram exhibited a higher AUC (Fig. 3D) than the clinical nomogram alone (Fig. 3C). Similar results were observed in the validation cohort (Fig. 3E, F), indicating that the discrimination ability of the clinical–radiomics nomogram was significantly better than that of the clinical nomogram and demonstrating the additional prognostic value of the Rad-score. The calibration curves of the clinical and clinical–radiomics nomograms (Fig. 4A, B) for the 1-, 3-, and 5-year OS demonstrated that the clinical–radiomics nomogram achieved better agreement between the predicted survival and the observed outcomes. The decision curves for OS prediction shown in Fig. 4C reflected the satisfactory clinical utility of the clinical–radiomics nomogram. In addition, the incorporation of the Rad-score with the clinical nomogram resulted in an NRI of 34.4% (95% CI: 0.029–0.506) and an IDI of 10% (95% CI: 0.020–0.199) (Fig. 4D), demonstrating improved prediction efficiency and classification accuracy for OS prediction.

**Table 3:** Univariate and multivariable Cox regression analyses of OS after definitive chemoradiotherapy in patients with proximal ESCA in the training cohort

Variables	Univariate cox regression		Multivariate cox regression	
	HR (95% CI)	*p* value	HR (95% CI)	*p* value
Age	1.031 (1.000–1.062)	0.048		
Gender	2.127 (1.140–3.968)	0.018	3.810 (1.866–7.779)	< 0.001
cN stage				
N0		0.063		
N1	0.812 (0.399–1.651)	0.566		
N2	1.866 (0.871–4.000)	0.109		
N3	1.525 (0.424–5.477)	0.518		
Treatment modality	0.307 (0.171–0.552)	< 0.001	0.141 (0.068–0.290)	< 0.001
NLR	1.055 (0.999–1.115)	0.054	1.085 (1.018–1.156)	0.013
AISI	1.000 (1.000–1.001)	0.074		
Rad-score	8.895 (2.775–28.506)	< 0.001	7.063 (2.190–22.774)	0.001

*NLR* neutrophil to lymphocyte ratio, *AISI* aggregate index of systemic inflammation, *Rad-score* radiomics score

### Association of the Rad-score with the TME

**Fig. 5:**
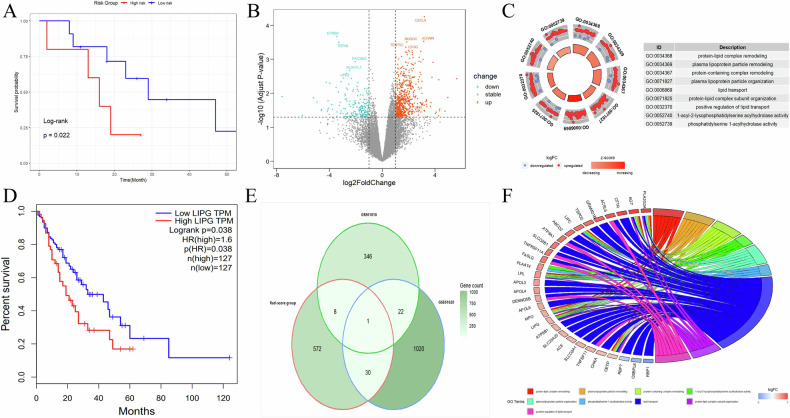
**A** Kaplan–Meier OS analysis curve of patients at different risks stratified by the Rad-score in the TCGA/TCIA cohort. **B** Volcano plot showed upregulated and downregulated genes in the high-score group. **C** GO enrichment analysis results of the DEGs in the high-score group. **D** Kaplan–Meier OS analysis curves of patients with high- and low-LIPG expression in the TCGA dataset. **E** Venn plot of common DEGs between different groups. **F** Chord diagram of GO enrichment analysis

Patients in the TCGA/TCIA cohort were separated into high-score and low-score groups on the basis of the Rad-score (14.460). The survival analysis yielded a consistent outcome, indicating that the high-score group had an unfavorable prognosis (*p* = 0.022) and confirming the validity of the Rad-score (Fig. 5A). A total of 797 DEGs were obtained, of which 611 DEGs were upregulated and 186 were downregulated in the high-score group (Fig. 5B). GO enrichment analysis of biological processes indicated that the DEGs were mainly enriched in lipid transport, protein-lipid complex subunit organization, and positive regulation of lipid transport (Fig. 5C). The differential analysis conducted in the GSE61816 and GSE61620 datasets revealed 377 and 1073 genes upregulated in the radiotherapy resistance group, respectively. Interestingly, when we attempted to explore the potential associations among the upregulated genes in these three groups, we ultimately found LIPG to be the core gene. Moreover, the Kaplan–Meier survival analysis in the TCGA-ESCA database indicated that patients with high LIPG expression had a much worse prognosis than those with low LIPG expression (*p* = 0.038; Fig. 5D). The distribution of the significant DEGs was assessed via a Venn diagram (Fig. 5E). The GO enrichment results showed that LIPG is primarily enriched in annotations related to lipid metabolism (Fig. 5F).

## Discussion

In this study, we developed a dual-region model that integrated intra- and peritumoral radiomics signatures using XGboost for survival prediction after dCRT in patients with proximal ESCA. Its performance surpassed all single-region radiomics signatures, demonstrating the complementary role of peritumoral radiomics features. We subsequently established and validated the incremental value of the radiomics nomogram in combination with clinical risk factors for the personalized estimation of OS. The results revealed that the combination of the Rad-score with the clinical nomogram significantly enhanced predictive accuracy and yielded a higher AUC and better calibration. Furthermore, by leveraging bioinformatics approaches, we found that lipid metabolic processes associated with radiotherapy resistance may be a potential biological mechanism for the risk stratification of patients with different Rad-score.

The significance of peritumoral radiomics features has been demonstrated in other cancers, such as non-small cell lung cancer [17], breast cancer [18], and cervical cancer [20], further suggesting that research on proximal ESCA should not be confined merely to the intratumoral area. To our knowledge, this study is the first to propose a radiomics prognostic model based on intra- and peritumoral radiomics features for proximal ESCA patients receiving dCRT. Our findings showed that peritumoral features exhibited excellent predictive performance and that the combination of intra- and peritumoral radiomics features had stronger predictive ability than the features from any single region, consistent with previous studies. Jain et al showed that peritumoral texture features are associated with a lower OS in small-cell lung cancer [21]. Meanwhile, most of the selected features were wavelet features, which were extracted at multiple scales through wavelet transformation multiscale analytical method based on raw images, reflecting the tumor heterogeneity in gray scales. Existing research has demonstrated significant correlations between wavelet features and underlying pathophysiological processes, proteomics profiles, and tumor morphology [22]. Consistent with the results of our study, Chen et al [23] also found that wavelet features are independent prognostic factors for hepatocellular carcinoma. Moreover, of the 11 radiomics features in the dual-region model, 8 features were derived from the peritumoral area, suggesting that the TME probably contains more prognostic information.

To explore the potential biological basis for the prognostic importance of radiomics features, we hypothesized that the high-score group indicates poorer prognosis through subtle changes in the TME that confer resistance to radiotherapy. Our findings demonstrated that LIPG is upregulated in the high-score group and it is also linked to radioresistance. LIPG, which belongs to the TG lipase family and is associated with lipid metabolism, probably modulates the generation, development, and prognosis of cancers by regulating lipid metabolism [24]. Similarly, the high-score group was associated with lipid metabolism-related functions, potentially indicating that lipid metabolism may play a crucial role in this population. In radiotherapy, reactive oxygen species-induced DNA damage and destruction of cellular structures like the cell membrane are the major mechanisms for eliminating tumor cells. In turn, tumor cells provide sufficient substrate and energy for damage repair through metabolic reprogramming, including lipid metabolism. One study found that FASN, an important enzyme in de novo palmitate synthesis, regulates PARP1 expression via the NF-κB pathway to confer radioresistance [25]. Additionally, tumor cells may adapt their lipid metabolism to mitigate radiation-induced ferroptosis by decreasing the abundance of ferroptosis-susceptible polyunsaturated fatty acids on their cell membrane [26]. Michelet et al revealed that high levels of free fatty acids in the TME cause lipid accumulation in natural killer cells and reduce their effector function, promoting an immune-suppressive TME [27]. Altogether, the reprogramming of lipid metabolism plays an essential role in the development of resistance to radiotherapy. Thus, pretreatment noninvasive identification of TME characteristics associated with an increased likelihood of radiotherapy resistance will offer novel insights to improve the efficacy of tumor therapy. Our research revealed a relationship between radiomics and metabolic patterns in the population showing radioresistance, which may have greater clinical value.

Our study still had several limitations. First, large-scale prospective studies are needed to further validate these conclusions in future studies since our study had a small sample size and was retrospective in nature. Second, only a small number of patients had both CT and genomic data from the TCGA cohort and they were all patients with ESCA, which does not completely fit the criteria for proximal ESCA. Third, the hypothesis for the radiomics-related biological basis was derived from the evaluation of the small cohort, which had limited statistical power, and additional validation in future cohorts with larger sample sizes may be required. Finally, the delineation of ROIs relies on manual intervention, the automatic segmentation should be adopted in future studies to reduce subjectivity.

In conclusion, the peritumoral radiomics features provided supplementary information about the TME for predicting the OS of patients with proximal ESCA. The predictive ability of the dual-region radiomics model outperformed that of the intratumoral or peritumoral models alone. Moreover, the dual-region radiomics nomogram provided adequate incremental value to the use of traditional clinical risk factors for individualized OS estimation. Our findings also indicated that radiomics features may reflect lipid metabolism related to radioresistance.

## Supplementary information

ELECTRONIC SUPPLEMENTARY MATERIAL

## Data Availability

Data generated or analyzed during the study are available from the corresponding author by request.

## Abbreviations

CT: Computed tomography
DEG: Differentially expressed gene
ESCA: Esophageal cancer
GBM: Gradient boosting machine
GO: Gene ontology
ICC: Interclass correlation coefficients
IDI: Integrated discrimination improvement
LASSO: Least absolute shrinkage and selection operator
NLR: Neutrophil–lymphocyte ratio
NRI: Net reclassification improvement
OS: Overall survival
PNI: Prognostic nutritional index
ROC: Receiver operating characteristic
ROI: Regions of interest
RSF: Random survival forest
SVM: Support vector machine
TCGA: The cancer genome atlas
TCIA: The cancer imaging archive
TME: Tumor microenvironment
VOI: Volume of interest
XGboost: Extreme gradient boosting
