# Supplementary material for: Machine learning-based radiomics prognostic model for patients with proximal esophageal cancer after definitive chemoradiotherapy
Source: Insights Imaging. 2024 Nov 29;15:284. doi: 10.1186/s13244-024-01853-y (PMC11607220; doi:10.1186/s13244-024-01853-y)
Supplement: Supplementary file 1 — ELECTRONIC SUPPLEMENTARY MATERIAL [file 13244_2024_1853_MOESM1_ESM.pdf]

Machine learning-based radiomics prognostic model for patients with proximal esophageal cancer after definitive chemoradiotherapy

ELECTRONIC SUPPLEMENTARY MATERIAL

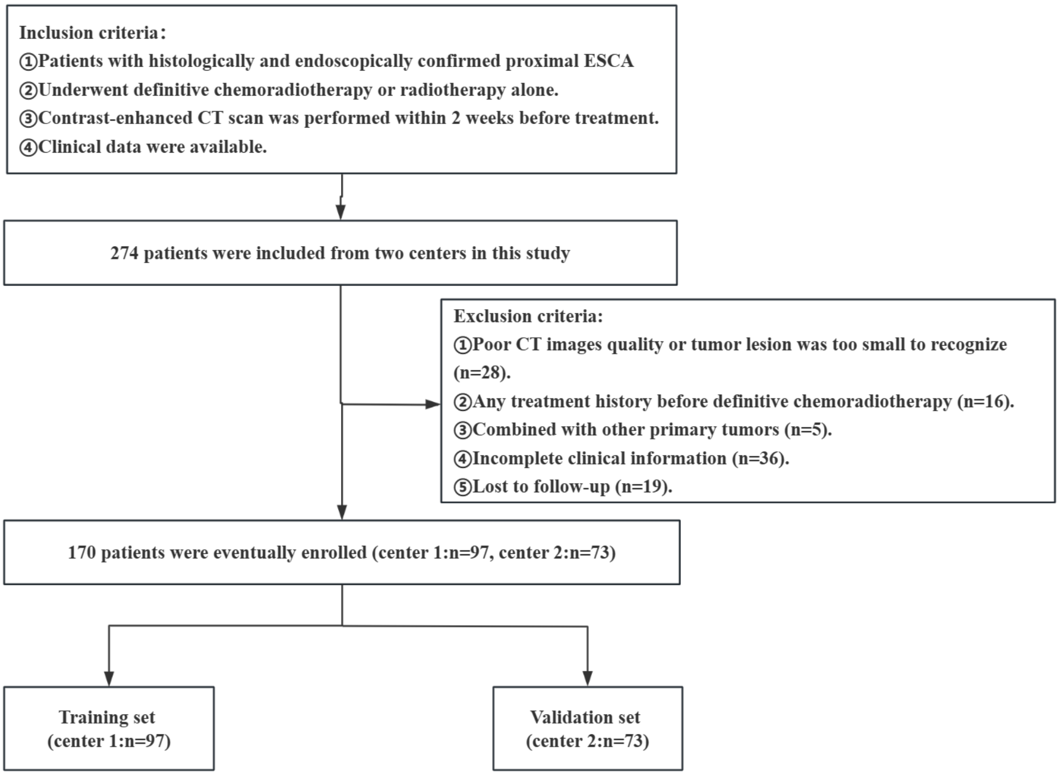

Supplementary Figure 1. Flow of patients selection according to inclusion and exclusion criteria.

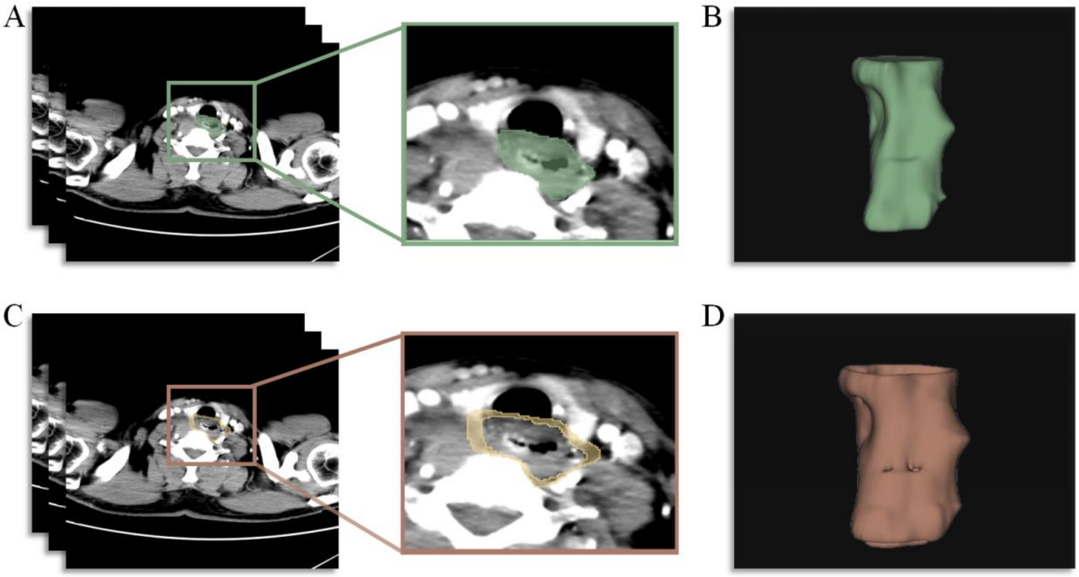

Supplementary Figure 2. Schematic diagram of lesion segmentation Arterial-phase CT images of a patient with proximal esophageal cancer. A: Intratumoral ROI (green markers) was outlined layer by layer; B: VOI of intratumoral regional fusion; C: 5 mm peritumoral ROI (red markers) was outlined layer by layer; D: VOI of peritumoral regional fusion.

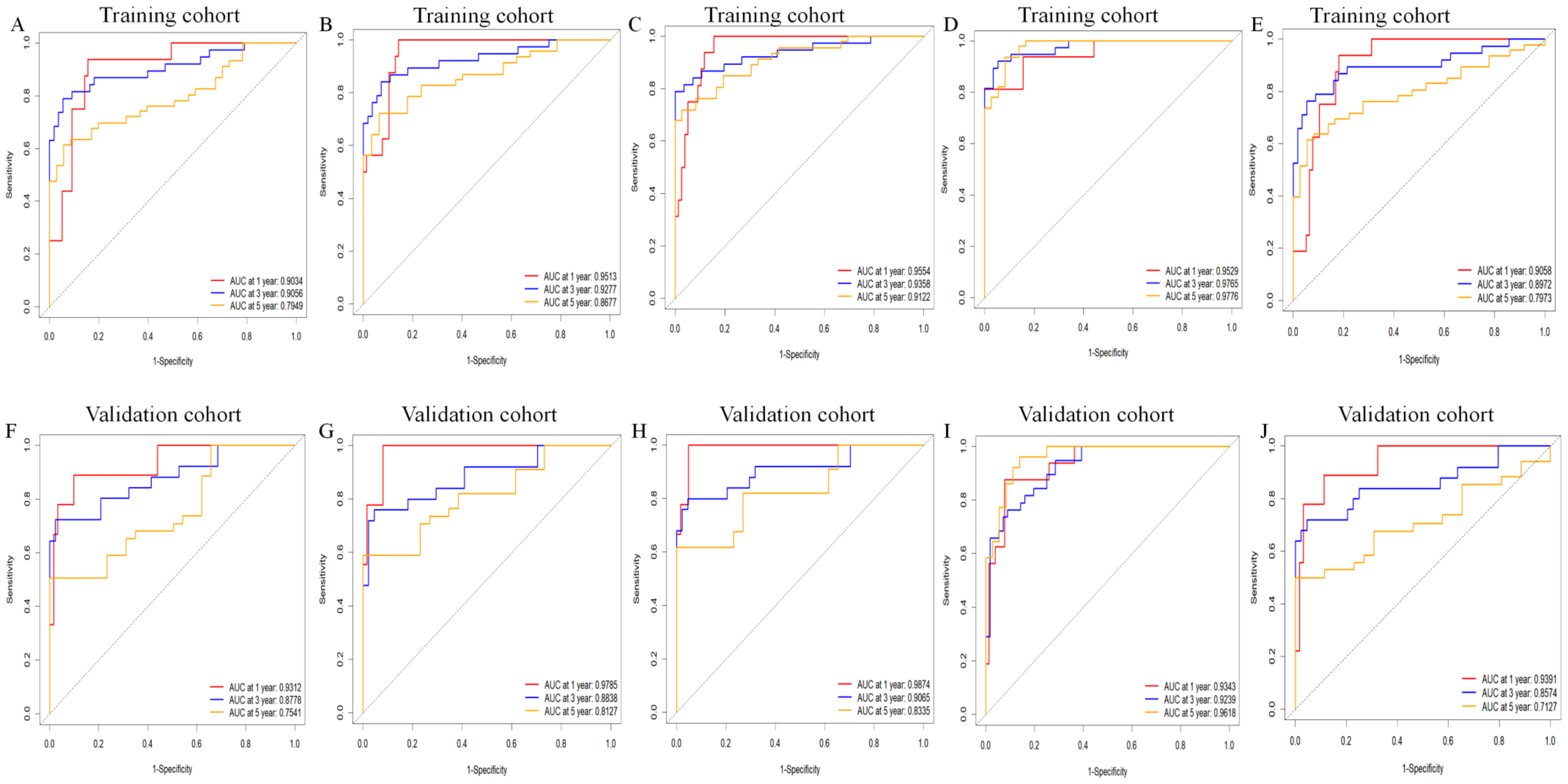

Supplementary Figure 3: The ROC curves of the Lasso (left 1), RSF (left 2), GBM (middle), XGboost (right 2), and SVM (right 1)in intra-tumoral region in the training (A-E) and validation (F-J) cohorts.

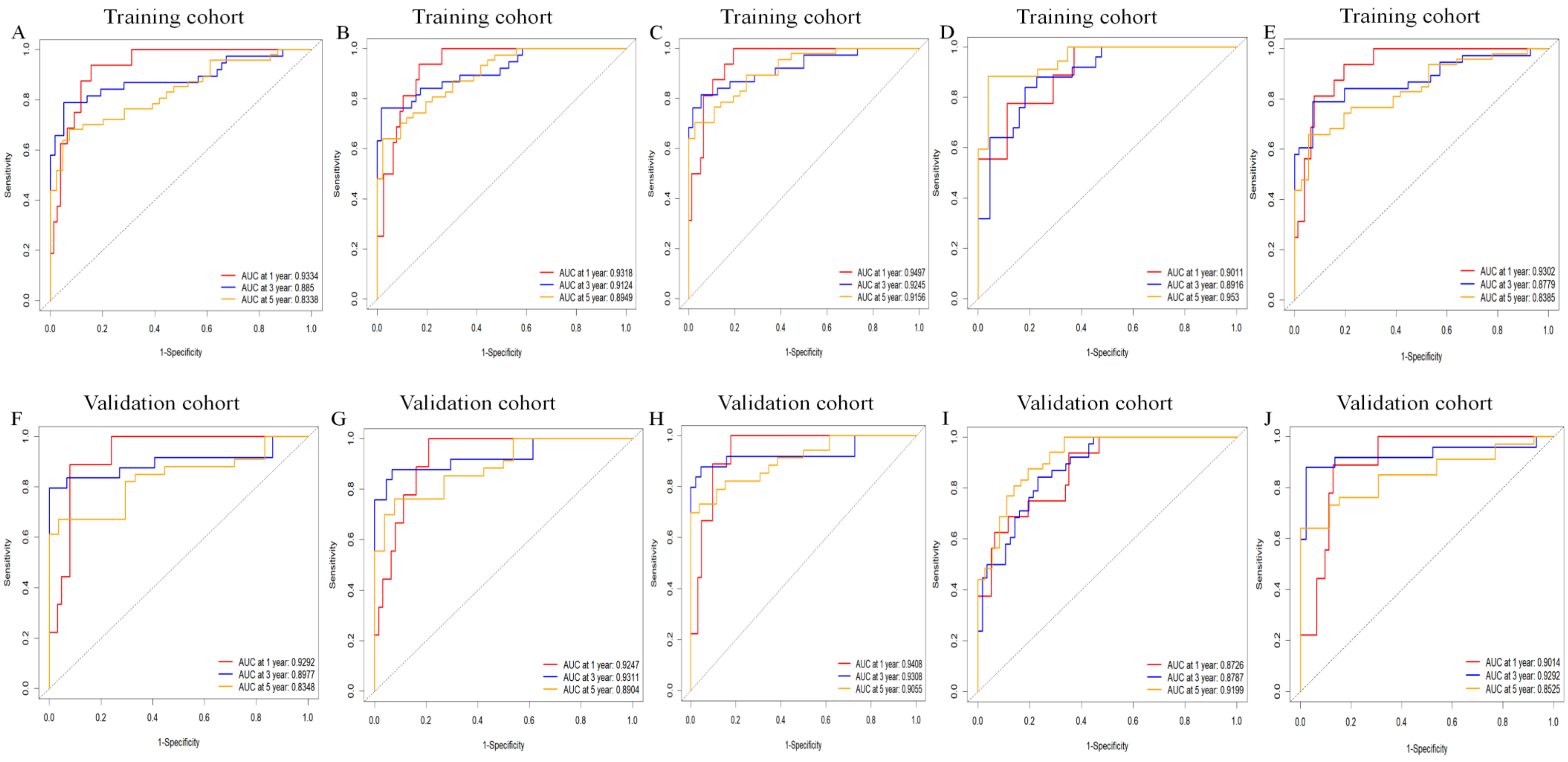

Supplementary Figure 4: The ROC curves of the Lasso (left 1), RSF (left 2), GBM (middle), XGboost (right 2), and SVM (right 1) in peri-tumoral region in the training (A-E) and validation (F-J) cohorts.

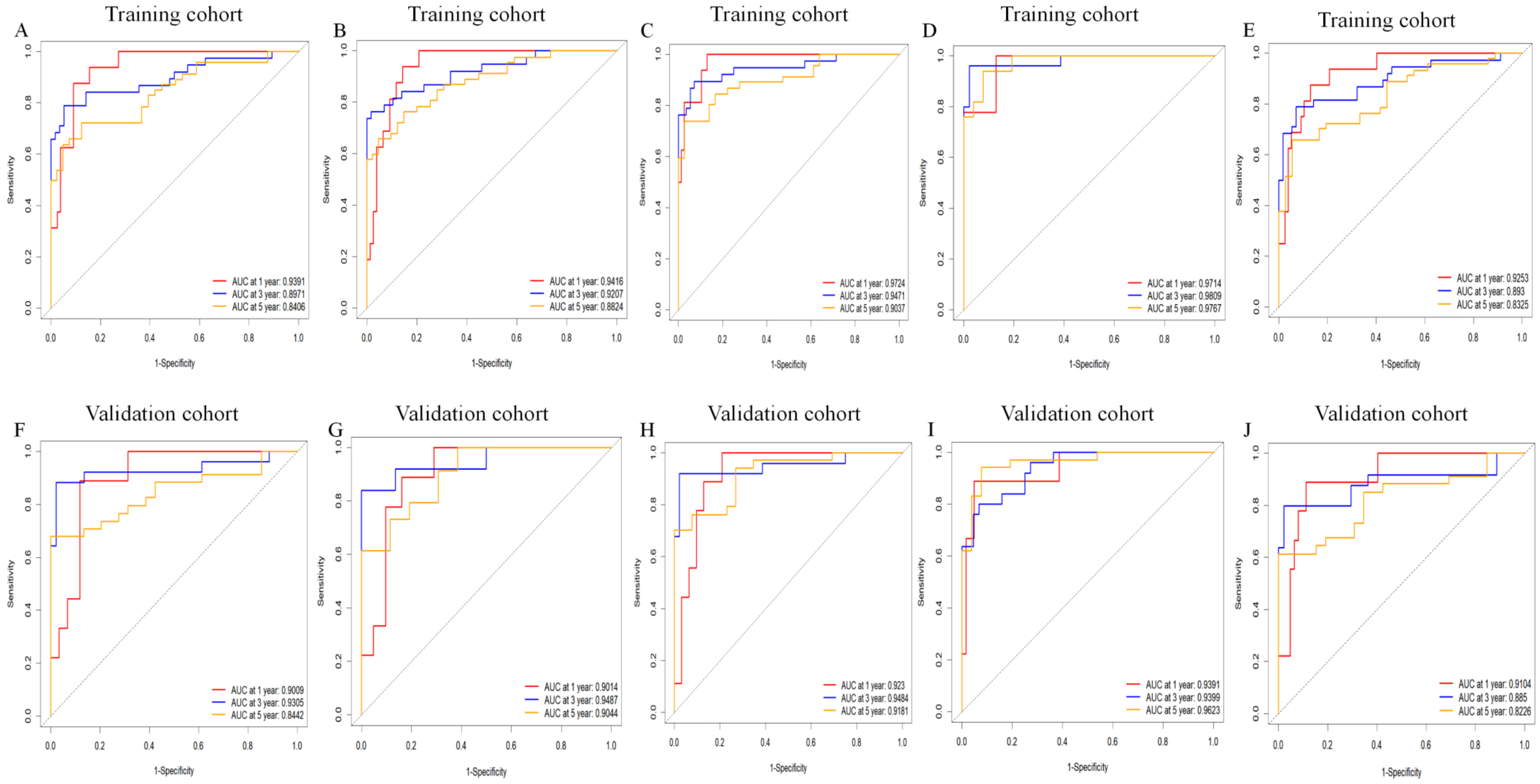

Supplementary Figure 5: The ROC curves of the Lasso (left 1), RSF (left 2), GBM (middle), XGboost (right 2), and SVM (right 1) in dual-region in the training (A-E) and validation (F-J) cohort

Supplementary Methods

Treatment and Follow-up management

Treatment protocol: Radiotherapy is the basic treatment regimen. A total dose of 50-64 Gy given in 25-32 fractions (2.0 Gy per fraction, 5 days per week) to the planning target volume (PTV) was delivered to the patients. Some patients were given an appropriate number of dislodges due to intolerance of adverse effects. For chemotherapy regimen, platinum-based chemotherapy combined with 5-fluorouracil or a taxane (docetaxel or paclitaxel) were administered on most patients, some patients who cannot tolerate platinum-based therapy were given single-agent capecitabine or S1.

Follow-up programmes: monthly for the first year after radiotherapy, and every 3 months for the second years, then every 6 months for 3-5 years, finnaly annually thereafter. Each evaluation included the physical examination, the blood test, the barium esophagram, the abdominal ultrasound, and the CT scan with coverage from the neck to the chest.

Regions of interest delineation and Feature extraction

To reduce the differences in scanning equipment and programmes, the arterial-phase enhanced CT images were first resampled to a voxel size of 1.0 × 1.0 × 1.0 mm^3 using B-spline interpolation sampling technique. Intratumoral delineation covered the whole tumor in all slices with the primary lesion present, the peritumoral region was annotated as the area where intratumoural ROIs flared out by 5 mm, which include the adjacent tissue and lymph nodes immediately around the esophagus and the airway, blood vessels, necrotic areas, vertebrae were excluded (A schematic of the lesion segmentation was shown in Supplementary Figure 2). The entire segmentation process described above was performed by two experienced radiologists (all with more than 10 years of experience in esophageal imaging) who were blinded to prognosis status. Reader 1 (Y.Z) was responsible for delineation and reader 2 (J.N.D) for

review. Moreover, we randomly selected a subset of 30 patients from the center 1 for the test-retest study. Their ROIs delineation was repeated by the same radiologist (Y.Z) and another radiologist (reader 3 who also with more than 10 years' experience in esophageal imaging, J.B), generating an intra-rater test dataset and an inter-rater test dataset. The ICCs were calculated to assess intra- and inter-observer agreement of features extraction, respectively. Inter- and intra-ICC greater than 0.75 indicated good consistency between reviewers. Finally, all ROIs were fused into three-dimensional VOIs which were used to extract radiomics features.

Statistical analysis

The K-S test, t-test, Mann-Whitney U-test, Chi-square test and Fisher's exact test were were conducted using SPSS software (version 26.0, IBM). Batch effect processing, standardise and normalise, intra- and inter-observer consistency, model construction, evaluation, and comparison were conducted using R software (version 3.6.3; https://www.r-project.org). The R packages included “scale”, “sva”, “psych”, “glmnet”, “randomForestSRC”, “gbm”, “xgboost”, “survivalsvm”, “rms”, “timeROC”, “rmda”, “ggDCA” and “survIDINRI”.

Supplementary Table 1: CT Imaging Acquisition

Parameters	Center 1	Center 2
Scanner	Discovery 750 (GE Healthcare, USA)	Discovery 750 (GE Healthcare, USA)
Tube voltage (kV)	120	120
Tube current (mA)	160	160
Contrast-enhanced CT	25 s after injection of contrast agent	25 s after injection of contrast agent
Contrast agent concentration	300 mgI/ml	300 mgI/ml
Contrast agent infused dose (ml/kg)	1.5	1.5
Contrast agent infused rate (ml/s)	3	3
layer thickness (mm)	5	5
